# Artificial Intelligence in Radiology: Augmentation, Not Replacement

**DOI:** 10.7759/cureus.86247

**Published:** 2025-06-17

**Authors:** Rohan Krishna NK, Anupama R.S., Shivaraj K

**Affiliations:** 1 Emergency Medicine, Fortis Hospital, Rajajinagar, Bengaluru, IND; 2 Internal Medicine, Fortis Hospital, Rajajinagar, Bengaluru, IND

**Keywords:** algorithmic bias, artificial intelligence, augmentation, clinical decision support, diagnostic imaging, human–machine collaboration, medical ethics, radiology

## Abstract

Artificial intelligence (AI) has rapidly become an influential presence in radiology, offering transformative possibilities in image interpretation, workflow optimization, and diagnostic support. Across healthcare systems worldwide, including resource-limited environments, these innovations hold promise for narrowing diagnostic and equity gaps. While concerns persist that AI may one day replace radiologists, this narrative oversimplifies the realities of clinical practice. Radiology is not merely about pattern recognition but also about clinical judgement, communication, and integration of complex patient data. In this context, AI should not be seen as a threat, but rather as an evolving tool with the potential to complement and enhance the radiologist's role. Current AI applications have already demonstrated considerable utility in well-defined areas. These include the detection of lung nodules, classification of breast lesions, and triage of acute stroke on CT imaging. By automating repetitive tasks and prioritizing critical findings, AI helps improve efficiency and reduce fatigue in high-volume settings. However, the integration of AI into routine clinical workflows brings several challenges that must be addressed. These include algorithmic biases due to non-representative training datasets, limited transparency in decision-making processes, and ethical questions around liability when errors occur. There is also a legitimate concern about over-reliance, particularly among less experienced clinicians, which could inadvertently erode critical thinking. Radiologists must remain at the forefront of AI development and implementation. Their clinical expertise is essential in designing, validating, and overseeing these tools to ensure that their use aligns with patient safety and care standards. AI should serve as an extension of human capabilities, not a replacement for the nuanced interpretation, empathy, and interdisciplinary collaboration that radiologists provide. This editorial explores both the potential and the pitfalls of AI in radiology, emphasizing the need for a balanced, collaborative approach. As the field moves toward greater digital integration, it is crucial to promote innovation that supports rather than diminishes the central role of human judgment. The future of radiology lies not in resisting technology but in shaping it, ensuring that the digital transformation enhances patient outcomes without compromising the human connection at the heart of medicine.

## Editorial

Artificial intelligence (AI) continues to transform medical imaging, where radiology has been one of its earliest and most active adopters. Deep learning algorithms are now capable of detecting patterns in medical images with remarkable speed and accuracy [1]. As AI continues to evolve, radiologists face an important question: Will it replace them, or will it empower them?

AI systems have already proved their usefulness in specific, well-defined activities. For example, deep learning models have performed as well as radiologists in recognizing lung nodules on low-dose CT images [2]. Beyond lung nodules, AI has shown promise in categorizing breast lesions with expert precision, detecting intracranial hemorrhages in emergency settings, and speeding up stroke triage via automated head CT interpretation [3,4]. Emerging studies across subspecialties, including emergency radiology, neuroradiology, and breast imaging, underscore how tailored AI pipelines can address the unique diagnostic and workflow challenges of each domain [5]. These results indicate that AI is no longer an aspiration; it is operational.

This advancement does not signal the end of radiologists but rather the evolution of their role. AI is increasingly integrated into clinical workflows to automate repetitive tasks such as organ segmentation, lesion detection, and report templating. By accelerating diagnostic throughput and prioritizing urgent findings, particularly in high-volume environments like emergency and oncologic imaging, AI helps reduce radiologist fatigue and supports timely clinical decision-making. This allows radiologists to redirect their expertise toward tasks that require nuanced clinical judgement, interdisciplinary collaboration, and patient engagement, all of which remain areas where human insight is essential [6].

A useful analogy is to think of AI in radiology as an autopilot in aviation. It is a remarkable aid that enhances performance, safety, and efficiency, but never replaces the need for a trained pilot. The autopilot can keep the plane steady, but when something goes wrong, such as turbulence, mechanical failure, or a complex emergency, human judgment becomes indispensable. Similarly, radiologists are essential for interpreting context, integrating clinical data, and making meticulous decisions that no algorithm can replicate.

Despite the promise, AI’s widespread adoption raises significant concerns. One major challenge is algorithmic bias. Most models are trained on datasets that may lack diversity in race, gender, or age, leading to reduced accuracy in underrepresented groups [7]. Another issue is the black-box nature of many AI tools. When a system makes a recommendation, it often cannot explain how it reached that conclusion. This lack of transparency undermines trust and complicates medico-legal accountability.

There have already been real-world consequences. For example, a widely used health management algorithm was found to significantly underestimate the health needs of Black patients compared to white patients due to its reliance on healthcare costs as a proxy for medical need [8]. Similarly, the Epic Sepsis Model, used in many U.S. hospitals, was found to miss a substantial proportion of actual sepsis cases while generating a high number of false alerts, partly due to its non-transparent and insufficiently validated design [9]. These cases sparked widespread concern about algorithmic fairness and equity, reinforcing the urgent need for diverse training datasets and clear governance frameworks [10].

Additionally, there is a real risk of overreliance. Without appropriate clinical oversight, AI outputs may be accepted at face value, particularly by trainees. This can lead to diagnostic errors, especially when tools are poorly validated or applied beyond their intended scope. Recent case-based analyses in neuroradiology, as highlighted in real-world examples published in peer-reviewed literature, have shown that AI tools, when misapplied or uncritically trusted, can result in misdiagnoses and delays in care. This underscores the irreplaceable value of human judgment in radiological practice [11].

It is also crucial to consider ethical and legal implications. If a diagnosis is missed due to a flawed algorithm, who bears responsibility? The radiologist who relied on the system? The software developer? Or the institution that deployed it? Clear governance frameworks must be established to guide such decisions.

Globally, AI is showing its utility not just in tertiary hospitals but also in resource-limited settings and partnership-led initiatives. For example, portable AI-powered ultrasound devices have been deployed in remote regions to assist non-specialist providers in making critical diagnostic decisions. Such tools help bridge the healthcare gap and offer a flexible solution to global radiologist shortages [12].

AI’s integration into radiology must be guided by three principles: transparency, collaboration, and education. Radiologists should be actively involved in the development and validation of AI tools. Hospitals and academic centers must ensure that these systems are tested on real-world data. Moreover, AI training should be included in radiology residency curricula, enabling future radiologists to interpret not only images but also the tools that help analyze them.

Rather than resisting this change, the radiology community must embrace it responsibly. We must ensure that AI complements rather than competes with our work. Radiologists remain central, not just for interpreting images but for understanding the patient behind the scan. AI may accelerate our work, but it cannot replace our reasoning, empathy, or clinical insight. The future of radiology will not be about humans versus machines, but about how both can coexist to deliver better patient outcomes.
